# An Online Dictionary Learning-Based Compressive Data Gathering Algorithm in Wireless Sensor Networks

**DOI:** 10.3390/s16101547

**Published:** 2016-10-22

**Authors:** Donghao Wang, Jiangwen Wan, Junying Chen, Qiang Zhang

**Affiliations:** School of Instrumentation Science and Opto-Electronics Engineering, Beihang University, Beijing 100191, China; dhwang@buaa.edu.cn (D.W.); woshicjy@126.com (J.C.); youleyuanzq@buaa.edu.cn (Q.Z.)

**Keywords:** wireless sensor networks, sparse representation, compressive sensing, data gathering, online dictionary learning

## Abstract

To adapt to sense signals of enormous diversities and dynamics, and to decrease the reconstruction errors caused by ambient noise, a novel online dictionary learning method-based compressive data gathering (ODL-CDG) algorithm is proposed. The proposed dictionary is learned from a two-stage iterative procedure, alternately changing between a sparse coding step and a dictionary update step. The self-coherence of the learned dictionary is introduced as a penalty term during the dictionary update procedure. The dictionary is also constrained with sparse structure. It’s theoretically demonstrated that the sensing matrix satisfies the restricted isometry property (RIP) with high probability. In addition, the lower bound of necessary number of measurements for compressive sensing (CS) reconstruction is given. Simulation results show that the proposed ODL-CDG algorithm can enhance the recovery accuracy in the presence of noise, and reduce the energy consumption in comparison with other dictionary based data gathering methods.

## 1. Introduction

Wireless sensor networks (WSNs) which are composed of lots of tiny, resource-constrained and cheap sensor nodes are self-organized networks. These nodes are always deployed in distributed mode to perform various applications, such as healthcare monitoring, transportation systems, industry service, and environmental measurement of humidity or temperature data [1]. In each case, efficient data gathering for target information is one of the primary missions.

In a typical scenario, WSNs have many ordinary sensor nodes and a base station named sink node. The ordinary nodes can only perform simple measurement and communication tasks since they are always equipped with limited power supply and most of the time, it is difficult to replace or recharge the battery. In contrast, the sink node is capable of performing complex operations since it is usually supplied with unlimited resources. Thus how to balance the energy consumption and develop energy efficient data collection protocols is still a research hotspot.

To reduce energy consumption for data gathering in WSNs, distributed source coding (DSC) [2] was proposed to compress the raw data between the ordinary nodes. DSC-based data collection protocols are composed of two important procedures. The first step is the collection of spatial-temporal correlation properties of the raw data. The second is a coding step which is based on the Slepian-Wolf coding theory. The coding process imposes no communication burden among sensor nodes, but the data correlation of the whole network must be calculated at the sink node before data collection, which results in a relatively high computational cost.

In recent years, compressive sensing has emerged as a new approach for signal acquisition, which can guarantee exact signal reconstruction with a small number of measurements [3]. Data compression and data collection are integrated into a single procedure in compressive sensing-based data gathering methods. Moreover, the high computational burdens are transferred to the base station. Finally, the incomplete data can be recovered by various complicated reconstruction algorithms at the sink node. Nevertheless, to ensure exact reconstruction, the key point is that the signals are required to be sparse in some base dictionary. Sparse representation expresses signals as sparse linear combinations of the basis. Therefore, dictionary learning for sparse signal representation is one of the core problems of compressive sensing.

The paper presents an ODL-CDG algorithm. The proposed algorithm aims to reduce the energy consumption for data gathering problem in WSNs. How to design ODL-CDG algorithm to be robust to environmental noise is also our objective. The main contributions of this paper can be summarized as follows:

(1) Inspired by the periodicity of nature signals, the learned dictionary is constrained with sparse structure where each atom is a sparse linear combination of the base dictionary. We first apply the sparse structured dictionary in the compressive data gathering process.
(2) The self-coherence of the learned dictionary is introduced as a penalty during the optimization procedure, which reduces the reconstruction error caused by ambient noise.
(3) In respect of the sparse structured dictionary ***D*** and the Gaussian observation matrix ***Φ***, it’s theoretically demonstrated that the sensing matrix ***P*** = ***ΦD*** meets the property of RIP with very high probability. What’s more, the lower bound of necessary number of measurements for exact reconstruction is given.
(4) With these consideration, the online dictionary learning algorithm is designed to improve the adaptability of data gathering algorithm for a variety of practical applications. The training data is gathered in parallel with the compressive sensing procedure, which reduces the enormous energy consumption.

The remainder of this paper is organized as follows: in Section 2, we review the previous works involving dictionary learning and energy-efficient data gathering problem in wireless sensor networks. Section 3 presents the mathematical formulation of the problem in detail. Section 4 demonstrates the RIP property of the sensing matrix. In Section 5, the optimized solution of our proposed ODL-CDG problem is given and the convergence property is also analyzed. In Section 6, the performances of the ODL-CDG algorithm are verified on synthetic datasets and the real datasets by experimental results. Finally, conclusions are drawn and future work proposed in Section 7.

## 2. Related Work

In the past few years, much effort has gone into designing data gathering techniques with the aim of reducing energy consumption in WSNs. Luo et al. [4] first proposed a complete design for compressive sensing-based data gathering (CDG) in large scale wireless sensor networks. In CDG, the sensor readings are assumed to be spatially correlated. Moreover, the communication cost is reduced and the load balance is achieved simultaneously. Liu et al. [5] introduced a novel compressed sensing method called expanding window compressed sensing (EW-CS) to improve recovery quality for non-uniform compressible signals. Shen et al. [6] proposed non-uniform compressive sensing (NCS) for signal reconstruction in the WSNs. The spatio-temporal correlation of the sensed data and the network heterogeneity were both taken into consideration in NCS, which leads to significantly less samples. In [7], the authors presented a quantitative analysis of the primary energy consumption of WSNs. They pointed out that the compressed sensing and distributed compressed sensing may act as energy efficient sensing approaches in comparison with other compression techniques.

The abovementioned compressive sensing-based data gathering methods can relieve the energy shortage problem and prolong network lifespan. However, these methods have limitations in that the signals are assumed only to be sparsely represented in a specified basis, etc., in a wavelet, Discrete Cosine Transform (DCT) or Fourier basis. Actually, a single predetermined basis may not be able to sparsely represent all types of signals, since there is a wide variety of applications for WSNs.

To adapt to signals of enormous diversity and dynamics, dictionary learning from a set of training signals has received lots of attention. The goal is to train a dictionary that can decompose the signals using a few atoms. The K-SVD [8] method is one of the well-known dictionary learning algorithms, which can lead to much more compact representation of signals. Duarte et al. [9] proposed to train the dictionary and optimize the sampling matrix simultaneously. The motivation is to make the mutual coherence between the dictionary and the projection matrix as minimal as possible. Christian et al. [10] presented a dictionary learning algorithm called IDL which made a trade-off between the coherence of the dictionary to the observations of signal class and the self-coherence of the dictionary atoms. To accelerate the convergence of K-SVD, an overcomplete dictionary was proposed in [11]. The authors suggested updating the atoms sequentially, thus leading to much better learning accuracy when compared with K-SVD. In [12], a new dictionary learning framework for distributed compressive sensing application was presented utilizing the data correlation between intra-nodes and inter-nodes, which resulted in improved compressive sensing (CS) performance 

However, the case where there is no access to the original data is not taken into account in the above work. What’s more, obtaining the full original data may be costly in wireless sensor networks. That is our motivation to learn the dictionary from a compressive sensing approach. Studer et al. [13] investigated dictionary learning from sparsely corrupted signals or compressed signals. In [14], the authors further extended the problem of compressive dictionary learning based on sparse random projections. The idea was coming from their previous paper [15], where the compressive K-SVD (CK-SVD) algorithm was proposed to learn a dictionary using compressive sensing measurements. Aghagolzadeh et al. [16] associated the spatial diversity of compressive sensing measurements without additional structural constraints on the learned dictionary, which guaranteed the convergence to a unique solution with high probability. 

Nevertheless, the environmental noise is not considered in the methods mentioned above. As analyzed in Section 3, the reconstruction error caused by environmental noise is positively correlated with the self-coherence of the learned dictionary. Thus, the self-coherence of the learned dictionary is added as a penalty term during the dictionary updating step. The novel dictionary is also imposed by structural constraints. 

## 3. Problem Formulation

In this section, we introduce the related issues in respect to dictionary learning and compressive sensing theory. The final form of ODL-CDG problem is formulated in detail. The main notations of the paper are summarized in Table 1.

### 3.1. Compressive Sensing

Compressive sensing (CS) theory builds on the surprising revelation that a sparse signal can be recovered from a much smaller number of sampling values. Let ***x*** ∈ **R***^N^* be the original signal vector, which denotes sensor readings gathered in wireless sensor networks. Suppose $\Phi \in {\mathbb{R}}^{M\times N}\left(M<N\right)$ is the measurement matrix with independent and identically distributed Gaussian entries and unit norm columns. Thus the lower-dimensional linear measurement vector ***y*** ∈ **R***^M^* can be obtained from the following standard measurement model:

(1) y=Φx + e

where ***e*** ~ ***N***(0, *σ*^2^) ∈ **R***^M^* is a white Gaussian noise vector.

Since sensor readings have spatial correlation, the signal vector ***x*** is assumed to be *K*-sparse in a given orthonormal basis ***Ψ*** = [*Ψ*_1_ *Ψ*_2_…*Ψ_N_*], *Ψ_i_* ∈ **R***^N^*. That is:

(2) x=Ψθ

where vector ***θ*** = [*θ*_1_,*θ*_2_,…*θ_N_*]^T^ is the corresponding sparse coefficients, with the constraint of ${\left\|\theta \right\|}_{0}=\;K\ll N$. The orthonormal basis ***Ψ*** can be constructed from various bases: DCT, wavelets, curvelets, etc.

As the number of equations *M* is much smaller than the number of variables *N*, the reconstruction of original signal ***x*** is an under-determined problem. An initial approach to solve the problem of recovering ***x*** depends on solving the following *l*_0_ minimization problem:

(3) $$\underset{\theta \in {\mathbb{R}}^{N}}{\min}{\left\|\theta \right\|}_{0}\;,\;s.t.\;{\left\|y\;-\;\Phi \Psi \theta \right\|}_{2}\leq \eta$$

where *η* is the expected noise on the measurements, ${\left\|•\right\|}_{0}$ denotes the number of nonzero entries of vector ***θ***, and ${\left\|•\right\|}_{2}$ counts the standard Euclidean norm. The above problem is NP-hard, so it’s numerically unstable to seek a global solution. To get an approximate solution, various greedy algorithms could be employed, like compressive sampling matching pursuit (CoSaMP) [17], Orthogonal Matching Pursuit with Replacement (OMPR) [18], and stagewise orthogonal matching pursuit (StOMP) [19].

Fortunately, the above problem is equivalent to the following *l*_1_ minimization problem under certain conditions. Thus the recovery can be obtained using linear programming (LP) techniques, searching for resolution of:

(4) $$\underset{\theta \in {\mathbb{R}}^{N}}{\min}{\left\|\theta \right\|}_{1}\;,\;s.t.\;{\left\|y\;-\;\Phi \Psi \theta \right\|}_{2}\leq \eta$$

If matrix ***P*** = ***ΦΨ*** satisfies the RIP [20], the solutions of optimization Equations (3) and (4) coincide with each other. The definition of RIP is as follows:

**Definition 1.** **(Restricted Isometry Property):** *Let **P** =* ***ΦΨ*** *be an M × N matrix and let **θ** be the sparse vector. The number of nonzero entries of vector **θ** is no larger than K. Define the K-restricted isometry constant δ_K_ as the smallest constant that satisfies:*

(5) $$\left(1-\delta_{K}\right){\left\|\theta \right\|}_{2}^{2}\leq {\left\|Ρ\theta \right\|}_{2}^{2}\leq \left(1+\delta_{K}\right){\left\|\theta \right\|}_{2}^{2}$$

*Then the matrix **P** =* ***ΦΨ*** *is said to satisfy the K-restricted isometry constant with the constant δ_K_*.

### 3.2. The Conventional Dictionary Learning Methods

Although exact recovery on account of a fixed sparse representation dictionary is guaranteed for inherently sparse signals or compressible signals, natural signals tend to have various types. Consequently, a single fixed dictionary would not be enough to sparsely represent all types of signals. Hence, much work has been done to achieve sparse redundant dictionary using dictionary learning methods, since it can enhance their ability to adapt to different types of signals.

Let ${\left\{x^{i}\right\}}_{i=1}^{L}$ denote training data for dictionary learning. ***x****^i^* ∈ **R***^N^* represents a data vector and *L* represents the amount of training data. Thus the data matrix ***X*** = [***x***^1^, ***x***^2^,…,***x****^L^*] ∈ **R***^N^*^×*L*^ is obtained. Then the general form of conventional dictionary learning methods can be rewritten as:

(6) $$\underset{D,C}{\min}{\left\|X-DC\right\|}_{F}^{2}\;,\;s.t.\;\forall i,\;{\left\|c_{i}\right\|}_{0}\leq S$$

where ‖•‖*_F_* represents matrix Frobenius norm, ***D*** ∈ **R***^N^*^×*K*^ denotes the sparse redundant dictionary, and ***C*** ∈ **R***^K^*^×*L*^ denotes the sparse matrix.

However, it is a fact that the original training data may not be available, or the cost for obtaining enough original data is high. In this paper, we are interested in training a sparse representation dictionary with only a few of CS measurements, which are linear projections of the original signals ***X*** onto a random sensing matrix ***Φ***. The problem of learning a dictionary ***D*** ∈ **R***^N^*^×*K*^ from a series of linear and non-adaptive measurements is defined as:

(7) $$y^{i}\;=\;\Phi Da^{i}，i\;=\;1,...,L$$

with ***y****^i^* ∈ **R***^M^* representing the compressed version of ***x****^i^* and ***a****^i^* representing the sparse column vector of the sparse matrix.

### 3.3. Sparse Structured Dictionary

In the sparse dictionary model ***D***, it is assumed that each atom of the dictionary can be expressed as a sparse linear combination of few atoms in a fixed base dictionary ***Ψ***. The dictionary is therefore expressed as:

(8) D=ΨΘ

where $\Theta \in R^{N\times p}$(*p* ≥ *N*) is the atom representation dictionary which is assumed to be sparse. Obviously, the base dictionary ***Ψ*** should contain some prior knowledge about the training signals. The matrix ***Ψ*** itself can also act as the sparse representation dictionary, such as the (DCT) dictionary or the overcomplete dictionary learned through other methods. The sparse dictionary model can adapt to various signals by modifying the matrix ***Θ***. As a general, with another ***Θ*** columns added to the base dictionary, the matrix ***Θ*** can be expressed as the following structure:

(9) $$\Theta \;=\;\left[I_{N\times N}|\Sigma_{N\times N}\right]$$

where ***Σ*** is assumed to be sparse and normalized.

### 3.4. The Recovery Error Penalty

In practical applications, the compressive data gathering procedure is corrupted with ambient noise. As shown in Equation (1), ***e*** represents the measurement noise. We employ the mean square error (MSE) to estimate the performance of reconstructing a sparse random vector ***θ*** in the presence of random Gaussian noise vector ***e***:

(10) $$\mathrm{MSE}\;=\;E_{\theta ,e}\left[{\left\|\hat{\theta }-\theta \right\|}_{2}^{2}\right]$$

where $\hat{\theta }$ denotes an estimation value of ***θ*** and *E_θ,e_*(•) denotes the mathematical expectation concerning the joint distribution of the random vectors ***θ*** and ***e***. The well-known oracle estimator assumes that the position of non-zero entries of sparse vector ***θ*** is known as a priori knowledge. The prior support is defined as a set Γ ⊂{1,2,…,*N*}. Thus Equation (1) can be expressed in the following form:

(11) $$y=\Phi DI_{\Gamma }\theta_{\Gamma }+e$$

where ***I*_Γ_** denotes the matrix obtained by just preserving the corresponding columns of the identity matrix on the support of Γ, and ***θ*_Γ_** ∈ **R***^S^* denotes the vector obtained by deleting the set of entries out of the support Γ. Then the formulation of the oracle estimator is given as follows [21]:

(12) $${\mathrm{MSE}}^{oracle}\;=\sigma^{2}Tr\left[{\left(I_{\Gamma }^{T}D^{T}\Phi^{T}\Phi DI_{\Gamma }\right)}^{-1}\right]$$

where *Tr*(•) denotes the trace of a matrix.

To mitigate the MSE caused by ambient noise, we consider making $Tr\left[{\left(I_{\Gamma }^{T}D^{T}\Phi^{T}\Phi DI_{\Gamma }\right)}^{-1}\right]$ as small as possible. As theoretically discussed in [22], the term $Tr\left[{\left(I_{\Gamma }^{T}D^{T}\Phi^{T}\Phi DI_{\Gamma }\right)}^{-1}\right]$ is positively correlated with the self-coherence of the sparse dictionary. Therefore, the penalty term $∥D^{T}D-I{∥}_{F}^{2}$ is introduced here to constrain the self-coherence of the learned dictionary.

### 3.5. The Final Form of ODL_CDG Problem

In this section, the method for training a sparse representation dictionary with only a few CS measurements is presented. The CS measurements can be arbitrary linear combinations of the original signals. The self-coherence of the learned dictionary is introduced to reduce the recovery error. Thus, in consideration of the sparse structured constraint and the self-coherence of the learned dictionary, the sparse dictionary model can be obtained from low dimensional CS measurements. Finally, the following optimization problem is obtained:

(13) $$\underset{A,\;D}{\min}\left\{\frac{1}{2}{\left\|Y-\Phi DA\right\|}_{F}^{2}+{\left\|D^{T}D-I\right\|}_{F}^{2}+\lambda_{A}{\left\|A\right\|}_{1}\right\}\;,\;s.t.\;{\left\|\Theta \right\|}_{1}\leq \varepsilon_{\theta }$$

## 4. Necessary Guarantees for Signal Reconstruction

Cai et al. [23] proved that Basis Pursuit algorithm could guarantee the reconstruction of Equation (4) in the following theorem:

**Theorem** **1.** *Assume that the measurement matrix* ***Φ** satisfies $\delta_{2s}\left(\Phi \right)<1/\sqrt{2}$ for some S∈ℕ. Let **θ** be a S-spare vector. Then, the recovery error of Equation (4) is bounded by the noise level.*

The reconstruction of signals that are sparse in an orthonormal basis is guaranteed by the above theorem. Nevertheless, we mainly stress the problem that signals are not sparse in an orthonormal basis but rather in a redundant dictionary ***D*** ∈ **R***^N×2N^*, as described in Section 3.2 and Section 3.3. For the sake of elaboration convenience, the following two lemmas are given:

**Lemma** **1.** *Let entries of* ***Φ*** ∈ **R***^M×N^ be independent normal variables with mean zero and variance n^−1^. Let **D***_Λ_*, i.e.,* |Λ| = S, *be the submatrix extracted from columns of the redundant matrix **D**. Define the isometry constant δ*_Λ_ = *δ*_Λ_*(**D**) and v: = δ*_Λ_ *+ δ + δ*_Λ_*δ for 0 < δ < 1. Then:*

(14) $$\left(1-\nu \right){\left\|\theta \right\|}_{2}^{2}\leq {\left\|\Phi D_{\Lambda }\theta \right\|}_{2}^{2}\leq \left(1+\nu \right){\left\|\theta \right\|}_{2}^{2}$$

*with probability exceeding:*

(15) $$1-2{\left(1+\frac{12}{\delta }\right)}^{S}e^{-\frac{c}{9}\delta^{2}M}$$

*where c is a positive constant, in particular c equals* 7/18 *for the Gaussian matrix* ***Φ****.*

**Proof.**  The proof of lemma 1 can be found in [24]. □

**Lemma** **2.** *The restricted isometry constant of a redundant dictionary **D** with coherence **μ** is bounded with:*

(16) $$\delta_{S}\left(D\right)\leq (S-1)\mu$$

**Proof.**  This can be obtained from the proof [25]. □

**Theorem** **2.** *Assume that the structure of our redundant dictionary is **D** =* ***ΨΘ** =* ***Ψ***[***I**_N×N_**Σ**_N×N_*], *where **Ψ** is the orthogonal base dictionary, i.e., discrete cosine transform (DCT) based ion* **R***^N^ for N =* 2^2*p*+1^. *The number of atoms is K =* 2^2*p*+2^. *Suppose that the sparsity of the signal is smaller than* 2^*p*−4^, *the necessary sampling number that could guarantee signal reconstruction is obtained by:*

(17) $$M\geq C_{1}\left(4S\left(2p\log2-\log S\right)+C_{2}+t\right)$$

*with the constants C*_1_ ≈ 524.33 *and C*_2_ ≈ 5.75*.*

**Proof.**  For t > 0, assume that the local isometry constant of matrix P = **ΦΨ** is δ_Λ_ (P), which is no larger than δ_Λ_ (D) + δ + δ_Λ_ (D)δ with probability at least 1−e^−t^. □

By Lemma 1, we obtain that:

$$\begin{array}{l} Ρ\left(\delta_{\Lambda }\left(Ρ\right)\;>\delta_{\Lambda }\left(D\right)+\;\delta +\delta_{\Lambda }\left(D\right)\delta \right)\\ \;\leq 2{\left(1+\frac{12}{\delta }\right)}^{S}e^{-\frac{c}{9}\delta^{2}M} \end{array}$$

Thus the global isometry constant $\delta_{S}\left(A\right)≔\underset{|\Lambda |=S}{\sup}\;\delta_{\Lambda }\left(A\right),\;S\epsilon N$ is bounded over all $\left(\begin{array}{c} K\\ S \end{array}\right)$ possibilities. So:

$$\begin{array}{l} Ρ\left(\delta_{S}\left(Ρ\right)\;>\delta_{S}\left(D\right)+\;\delta +\delta_{S}\left(D\right)\delta \right)\\ \;\leq 2\left(\begin{array}{l} K\\ S \end{array}\right){\left(1+\frac{12}{\delta }\right)}^{S}e^{-\frac{c}{9}\delta^{2}M} \end{array}$$

Using the Stirling formula, and confining the above term to less than *e*^−*t*^, the following inequation is obtained:

2(eKS)S(1+12δ)Se−c9δ2M<e−t⇒2(KS)S(e(1+12δ))S<e−t+c9δ2M⇒log2+Slog(KS)+Slog(e(1+12δ))<−t+c9δ2M⇒c9δ2M≥Slog(KS)+Slog(e(1+12δ))log2+t⇒M≥9δ−2c−1(Slog(KS)+Slog(e(1+12δ))+log2+t)⇒M≥9δ−2c−1(Slog(KS)+log(2e(1+12δ))+t)⇒M≥C1(Slog(KS)+C2+t)

The above theoretical derivation states that *δ_S_*(***P***) is less than *δ_S_*(***D***) + *δ* + *δ_S_*(***D***)*δ* with probability at least 1−*e*^−*t*^ when M≥C1(Slog(KS)+C2+t).

Let *μ* be the coherence of dictionary ***D***, we assume:

(18) $$S-1\leq \frac{1}{10}\mu^{-1}$$

Then combining with Lemma 2, we can obtain:

(19) $$\delta_{S}\left(D\right)\leq (S-1)\mu \leq \frac{1}{10}$$

Thus, defining *δ* = 7/33 yields:

(20) $$\begin{array}{l} \delta_{S}\left(Ρ\right)\;\leq \delta_{S}\left(D\right)+\;\delta +\delta_{S}\left(D\right)\delta \\ \;\leq \frac{1}{10}+\frac{7}{33}\left(1+\frac{1}{10}\right)=\frac{1}{3} \end{array}$$

As demonstrated by Theorem 1, the necessary number of samples to have $\delta_{2s}\left(\Phi \right)<1/\sqrt{2}$ is:

(21) M≥C1(Slog(KS)+C2+t)

Replacing *S* in Equation (21) with 2*S*, and plugging *K* = 2^2*p*+2^ and *δ* = 7/33 into Equation (21), the necessary number of samples is finally available. That is:

(22) $$M\geq C_{1}\left(2S\left(2p\log2+\log2-\log S\right)+C_{2}+t\right)$$

## 5. The Solution of the ODL-CDG Algorithm

Considering the demonstration in Section 4, we conclude that the ODL-CDG algorithm is feasible with a sufficient number of measurements. To get the solution by existing methods, the optimization Problem (13) is reformulated as an unconstrained optimization problem. Therefore, the cost objective function of final form of ODL-CDG problem is:

(23) $$\underset{A,\;\Theta }{\min}\left\{\frac{1}{2}{\left\|Y-\Phi \Psi \Theta A\right\|}_{F}^{2}+{\left\|{\left(\Psi \Theta \right)}^{T}\left(\Psi \Theta \right)-I\right\|}_{F}^{2}+\lambda_{A}{\left\|A\right\|}_{1}+\lambda_{\Theta }{\left\|\Theta \right\|}_{1}\right\}\;$$

where *λ*_A_ and *λ**_Θ_*** represent the sparse coefficient regularization parameter and the structured dictionary regularization parameter, respectively. The ODL-CDG algorithm is solved in a two-step iterative approach, which alternates between sparse coding and dictionary update procedures.

### 5.1. Sparse Coding

In the sparse coding step, sparse coefficients are obtained using the dictionary ***D*** which is computed from the previous step:

(24) $$\underset{A}{\min}\left\{\frac{1}{2}{\left\|Y-\Phi \Psi \Theta A\right\|}_{F}^{2}+\lambda_{A}{\left\|A\right\|}_{1}\right\}$$

The above optimization Problem (24) can be successfully computed using Matching Pursuit LASSO (MPL) [26]. MPL can greatly speed up the convergence of the Problem (24) when employed in batch-mode.

### 5.2. Dictionary Update

In the dictionary update step, with the consideration of the sparsity constraint on the dictionary, the following optimization equation is obtained:

(25) $$\underset{\Theta }{\min}\left\{\frac{1}{2}{\left\|Y-\Phi \Psi \Theta A\right\|}_{F}^{2}+{\left\|{\left(\Psi \Theta \right)}^{T}\left(\Psi \Theta \right)-I\right\|}_{F}^{2}+\lambda_{\Theta }{\left\|\Theta \right\|}_{1}\right\}\;$$

The object function in Equation (25) can be rewritten into the following form:

(26) $$\underset{\Theta }{\min}\;F(\Theta )\;=\;f(\Theta )\;+\;g(\Theta )$$

with:

(27) $$f(\Theta )\;=\;\frac{1}{2}{\left\|Y-\Phi \Psi \Theta A\right\|}_{F}^{2}+{\left\|{\left(\Psi \Theta \right)}^{T}\left(\Psi \Theta \right)-I\right\|}_{F}^{2}\;\mathrm{and}\;g(\Theta )\;=\;\lambda_{\Theta }{\left\|\Theta \right\|}_{1}$$

Obviously, the accelerated proximal gradient (APG) approach [27,28] can be used to solve the above unconstrained non-smooth convex problem, where both *f*, and *g* are convex. Furthermore, *f* is Lipschitz continuous:

(28) $${\left\|\nabla f(\Theta_{1})-\nabla f(\Theta_{2})\right\|}_{2}\leq L_{f}{\left\|\Theta_{1}-\Theta_{2}\right\|}_{2},\forall \Theta_{1}，\;\Theta_{2}\in {\mathbb{R}}^{N\times p}$$

where ‖•‖_2_ denotes the standard Euclidean norm and *L_f_* > 0 is the Lipschitz constant of ∇*f*.

Since *g*(***Θ***) is nonsmooth, it is difficult to directly minimize the objective function *F*(***Θ***). Instead, *F*(***Θ***) is approximated locally as a quadratic function at point ***Y_k_*** and we try to repeatedly solve:

(29) $$\Theta_{k+1}=\arg\underset{\hat{\Theta }\in {\mathbb{R}}^{N\times p}}{\min}Q(\Theta ,Y_{k})≐f(Y_{k})+\langle \nabla f(Y_{k}),\Theta -Y_{k}\rangle +\frac{L_{f}}{2}{{\left\|\Theta -Y_{k}\right\|}_{F}}^{2}+g(\Theta )$$

For convenience, let *S*_λ_(*y*) denote the soft-thresholding operator [29], which is defined as follows:

(30) $$S_{\lambda }(y)=\left\{ \begin{array}{l} y-\varepsilon ,\;\mathrm{if}\;y>\varepsilon \\ y+\varepsilon ,\;\mathrm{if}\;y<-\varepsilon \\ 0,\;\mathrm{otherwise} \end{array}\right.$$

where *y* ∈ **R** and ε > 0. This operator can be also useful when applied elementwise to vectors and matrices.

Let *G* ∈ **R***^N^*^×*p*^. Then:

(31) $$S_{\lambda_{\Theta }}(G)=\arg\underset{\hat{\Theta }\in {\mathbb{R}}^{N\times p}}{\min}\left\{\frac{1}{2}{{\left\|\Theta -G\right\|}_{F}}^{2}+\lambda_{\Theta }{\left\|\Theta \right\|}_{1}\right\}$$

where $S_{\lambda_{\Theta }}\left(G\right)$ is the soft-thresholding operator, as defined in Equation (30).

**Proposition** **1.** Let ***Y_k_*** ∈ **R***^N^*^×*p*^, *g*(***Θ***) = *λ_**Θ**_*‖***Θ***‖_1_. Assume *f*(***Θ***) is Lipschitz continuous with a positive constant *L_f_*, then we have:

(32) $$\arg\underset{\hat{\Theta }\in {\mathbb{R}}^{N\times p}}{\min}Q(\Theta ,Y_{k})=\;S_{\lambda {L_{f}}^{-1}}(G_{k})$$

where $G_{k}=Y_{k}-\frac{1}{L_{f}}\nabla f\left(Y_{k}\right)$.

**Proof.**  (33) $$\begin{array}{ll} Q(\Theta ,Y_{k}) & ≐f(Y_{k})+\langle \nabla f(Y_{k}),\Theta -Y_{k}\rangle +\frac{L_{f}}{2}{{\left\|\Theta -Y_{k}\right\|}_{F}}^{2}+g(\Theta )\\ & =f(Y_{k})+\langle \nabla f(Y_{k}),\Theta -Y_{k}\rangle +\frac{L_{f}}{2}{{\left\|\Theta -Y_{k}\right\|}_{F}}^{2}+\lambda_{\Theta }{\left\|\Theta \right\|}_{1}\\ & =\frac{L_{f}}{2}{{\left\|\Theta -\left(Y_{k}-\frac{1}{L_{f}}\nabla f(Y_{k})\right)\right\|}_{F}}^{2}-\;\frac{1}{2L_{f}}{\left\|\nabla f(Y_{k})\right\|}_{F}^{2}\;+\;f(Y_{k})+\;\lambda_{\Theta }{\left\|\Theta \right\|}_{1}\\ & =\frac{L_{f}}{2}{{\left\|\Theta -G\right\|}_{F}}^{2}+\lambda_{\Theta }{\left\|\Theta \right\|}_{1}+f(Y_{k})\;-\;\frac{1}{2L_{f}}{\left\|\nabla f(Y_{k})\right\|}_{F}^{2} \end{array}$$

□

Then combined with Equation (32), the final solution is obtained:

(34) $$\begin{array}{ll} \Theta_{k+1} & \;=\;\arg\underset{\hat{\Theta }\in {\mathbb{R}}^{N\times p}}{\min}Q(\Theta ,Y_{k})\\ & =\;\underset{\hat{\Theta }\in {\mathbb{R}}^{N\times p}}{\arg\;\min}\left\{\frac{L_{f}}{2}{{\left\|\Theta -G\right\|}_{F}}^{2}+\lambda_{\Theta }{\left\|\Theta \right\|}_{1}+f(Y_{k})\;-\;\frac{1}{2L_{f}}{\left\|\nabla f(Y_{k})\right\|}_{F}^{2}\right\}\\ & =\;\underset{\hat{\Theta }\in {\mathbb{R}}^{N\times p}}{\arg\;\min}\left\{\frac{L_{f}}{2}{{\left\|\Theta -G\right\|}_{F}}^{2}+\lambda_{\Theta }{\left\|\Theta \right\|}_{1}\right\}\\ & =\;S_{\lambda {L_{f}}^{-1}}(G_{k}) \end{array}$$

However, it is not always easy to compute the Lipschitz constant *L_f_*. The APG algorithm with a backtracking stepsize rule is employed in Algorithm 1. We appoint an initial estimate value of *L_f_* and increase the estimate gradually until the violation rule is reached.

Finally the pseudo-code of the ODL-CDG algorithm is shown in Algorithm 2.
**Algorithm 1.** APG with backtracking.**Initialization**: Let *L*_0_ > 0, *η* > 1**While** not converged **do**1: Find the smallest nonnegative interger *i_k_* with $\bar{L}=\eta^{i_{k}}L_{k-1}\left(k\geq 1\right)$ to satisfy     $F\left(S_{\lambda \bar{L}}\left(G_{k}\right)\right)\leq Q\left(S_{\lambda \bar{L}}\left(G_{k}\right),Y_{k}\right)$
2: Set $L_{k}=\eta^{i_{k}}L_{k-1}\left(k\geq 1\right)$ and compute    $Y_{k}\leftarrow X_{k}+\frac{t_{k-1}-1}{t_{k}}\left(X_{k}-X_{k-1}\right)$    $\Theta_{k+1}\leftarrow arg\;\underset{X}{\min}Q_{L_{f}}\left(\Theta ,Y_{k}\right)$    $t_{k+1}\leftarrow \frac{1+\sqrt{1+4t_{k}^{2}}}{2}$    k←k+1**End While**
**Algorithm 2.** ODL-CDG Algorithm.**Input: *Y,Φ,Ψ,****λ*_A_,*λ**_Θ_***,*T,ε_stop_***Output: *D*,**$\hat{X}$**Main procedure:****While**
*t* < *T* and $∥\Theta_{k+1}-\Theta_{k}{∥}_{2}>\varepsilon_{stop}$
**do**Sparse Coding using MPL:$\underset{A^{\left(t\right)}}{\min}\left\{\frac{1}{2}∥Y-\Phi \Psi \Theta^{\left(t-1\right)}A^{\left(t\right)}{∥}_{F}^{2}+\lambda_{A}∥A^{\left(t\right)}{∥}_{1}\right\}$Dictionary Update using APG (see Algorithm 1)$\underset{{\hat{\Theta }}^{\left(t\right)}}{\min}Q(\Theta ,Y)\;=\;\underset{\hat{\Theta }\in {\mathbb{R}}^{N\times p}}{\arg\;\min}\left\{\frac{L_{f}}{2}{{\left\|\Theta^{\left(t\right)}-G^{\left(t-1\right)}\right\|}_{F}}^{2}+\lambda_{\Theta }{\left\|\Theta^{\left(t\right)}\right\|}_{1}\right\}$t←t + 1**End while**Then $D\;=\;\Psi \left[I_{N\times N}|\Sigma_{N\times N}\right]=\;\left[\Psi \;\Psi \Sigma \right]$Compute $\hat{A}$: $\underset{\hat{A}}{\min}\left\{\frac{1}{2}{\left\|Y-\Phi D\hat{A}\right\|}_{F}^{2}+\lambda_{A}{\left\|\hat{A}\right\|}_{1}\right\}$$\hat{X}=D\hat{A}$

### 5.3. Convergence Analysis

As described above, the ODL-CDG algorithm contains the sparse coding step and the dictionary learning step. In the sparse coding step, the convergence of the optimization Problem (24) is guaranteed by MPL [26]. Furthermore, in the dictionary update step, the sequence of function values *F*(***Θ**_k_*) produced by APG is non-increasing, since the Lipschitz constant *L_f_* satisfies *L*_0_ ≤ *L_f(k)_* ≤ *ηL_f_* for every *k* ≥ 1. The convergence rate of APG with the backtracking rule is demonstrated as *O(k*^−2^), that is to say *F*(***Θ**_k_*) − *F*(Θ*) ≤ *Ck*^−2^ [27]. What’s more, the convergence of the alternating minimization method is also studied in [11]. Therefore, the convergence of ODL-CDG algorithm can be guaranteed.

## 6. Simulation

This section presents our simulation results on synthetic data and the real datasets. The performance of the proposed dictionary is compared with a pre-specified dictionary, like the DCT dictionary, and other dictionary learning approaches, such as K-SVD, IDL and CK-SVD.

### 6.1. Recovery Accuracy

The initial basis ***Ψ*** is a 50 × 50 DCT matrix. A set of training signals ${\left\{y_{i}\right\}}_{i=1}^{L}$ is generated by a linear combination of the original synthetic data. The process is accomplished by applying a projection matrix ***Φ*** with independent and identically distributed Gaussian entries and column normalization. Input parameters to Algorithm 1 are *λ*_A_ = 0.1, *λ**_Θ_*** = 0.05, *ε_stop_* = 0.001 and *T* = 100. 

**Figure 1:**
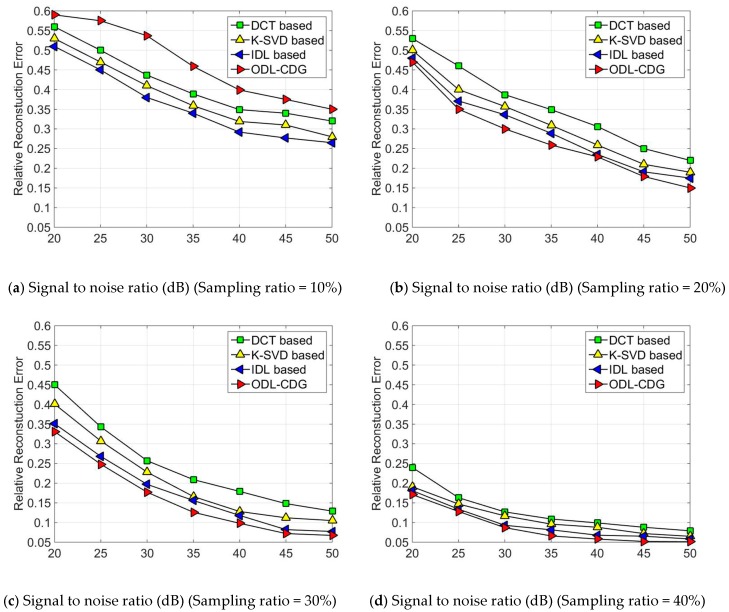
The relative reconstruction error of DCT, K-SVD, IDL and ODL-CDG under different signal-to-noise ratio: (**a**) Sampling Ratio = 10%; (**b**) Sampling Ratio = 20%; (**c**) Sampling Ratio = 30%; (**d**) Sampling Ratio = 40%.

**Figure 2:**
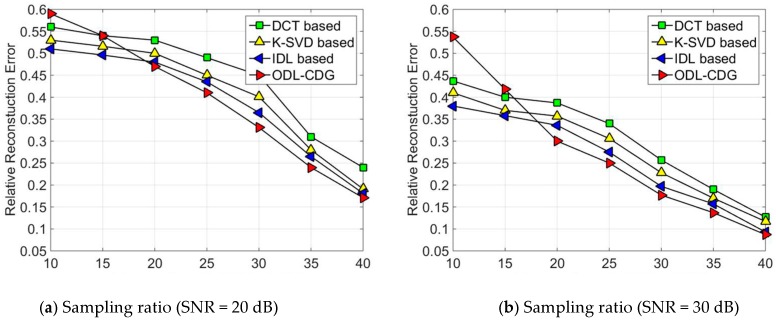

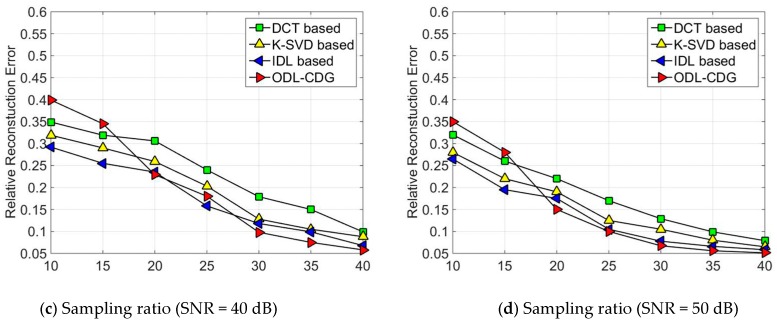
The relative reconstruction error of DCT, K-SVD, IDL and ODL-CDG under different sampling ratio: (**a**) SNR = 20 dB; (**b**) SNR = 30 dB; (**c**) SNR = 40 dB; (**d**) SNR = 50 dB.

The performance is evaluated by using the relative reconstruction error, i.e., $\frac{∥\hat{X}-X{∥}_{F}}{∥X{∥}_{F}}$, where ***X*** and $\hat{X}$ denote the original signal and the reconstructed signal, respectively. Each setting is averaged for 50 trials. The simulation results are presented in Figure 1 and Figure 2. In Figure 1, each subgraph corresponds to a certain amount of sampling ratio. The signals are added with white Gaussian noise, which yields the signal-to-noise ratio (SNR) to range from 20 dB to 50 dB. As can been seen from Figure 1a, ODL has poor performance when the sampling ratio is low, but the ODL dictionary outperforms both the DCT dictionary and the K-SVD method in the relative reconstruction error, when the sampling ratio is high (high than 20%). The fixed dictionary, DCT, is the worst case. That’s because the DCT dictionary using a fixed structure cannot sparsely represent synthetic data of various diversities. In comparison, K-SVD is better than the DCT dictionary. This is because K-SVD can adapt to sparsely represent the synthetic data by training. Since the IDL algorithm trains the dictionary using the self-coherence constraint term, the relative reconstruction error is smaller than DCT and K-SVD. Similar results can be obtained from Figure 2, where the relative reconstruction errors of DCT, K-SVD, IDL and ODL are obtained with sampling ratios of 10%, 15%, 20%, 25%, 30% and 40%, respectively. As we can see in our simulations, the results of ODL are much worse than DCT, K-SVD, and IDL when the sampling ratios is quite low (less than 10%). But ODL outperforms these algorithms compared by gradually increasing the sampling ratio.

### 6.2. Impact of Regularization Parameters on Sparse Representation Error

The performance of ODL-CDG algorithm may also be highly influenced by the setting value of regularization parameters *λ*_A_ and *λ_**Θ**_*. In this experiment, we analyze how the selected regularization parameters affect the sparse representation error. The optimal parameters for ODL-CDG algorithm is determined. The datasets used in this section are collected from the IntelLab [30]. We select the temperature values and the humidity values of size 54 × 100 between 28 February and 27 March 2004. The time interval is 31 s. We first solve the following sparse representation problem on training data ${\left\{x^{i}\right\}}_{i=1}^{L}$:

(35) $$\hat{\theta }=\underset{\theta \in {\mathbb{R}}^{L}}{\arg\min}{\left\|D\hat{\theta }-x^{i}\right\|}_{2}\;,\;s.t.\;{\left\|\theta \right\|}_{0}\leq S$$

where *S* denotes the sparsity of the coefficient ***θ***. 

**Figure 3:**
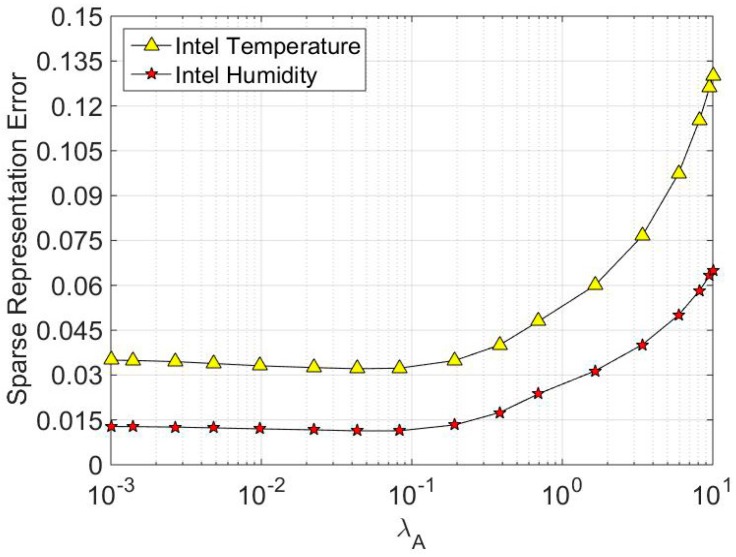
The impact of sparse coefficient regularization parameter *λ*_A_ on sparse representation error.

**Figure 4:**
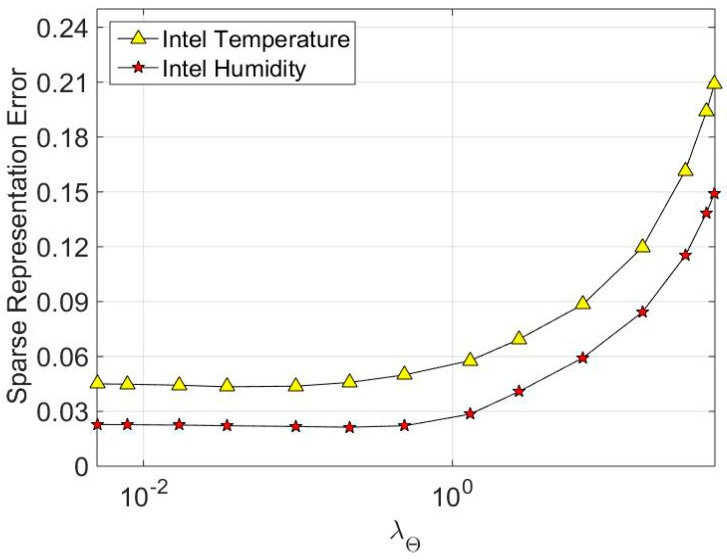
The impact of structured dictionary regularization parameter *λ**_Θ_*** on sparse representation error.

Then, the sparse representation error of the learned dictionary is evaluated using the root mean square error (RMSE), which is defined as follows:

(36) $$RMSE=\frac{1}{\sqrt{L}}\sum_{i=1}^{L}{\left\|D\hat{\theta }-x^{i}\right\|}_{2}$$

where *L* is the amount of training data. We average the experiment 50 times for every training data vector. Figure 3 shows the simulation results. In general, the sparse representation error is becoming larger as the parameter *λ*_A_ gradually increases. That’s because regularization parameter *λ*_A_ determines the sparsity of the sparse coefficient. To constrain the coefficients to be sparser, we need to increase *λ*_A_ to a specific threshold. However, as can be seen from Figure 3, the sparse representation error increases tremendously as *λ*_A_ exceeds 0.1. In Figure 4, parameter *λ***_Θ_** shows that it has the same trend as *λ*_A_ in impacting the sparse representation error. Based on the above discussion, we set the regularization parameter as a relatively small value, such as *λ*_A_ = 0.1 and *λ***_Θ_** = 0.05. These are also the optimal parameter values we set in Section 6.1. 

### 6.3. Energy Consumption Comparison

**Figure 5:**
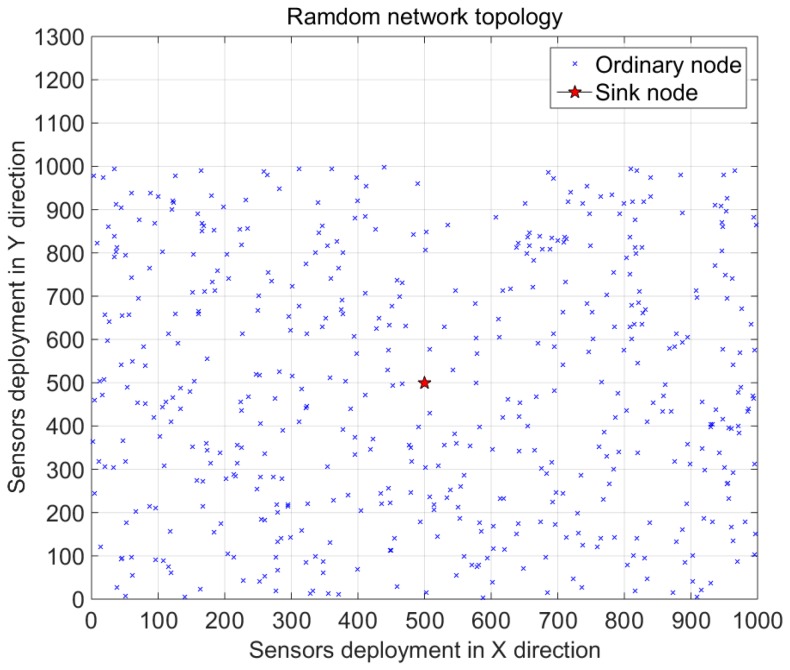
Random deployment of 500 sensor nodes.

In this subsection, we simulate the energy consumption of the ODL-CDG algorithm. The simulation platform is MATLAB. Suppose 500 nodes are randomly deployed in a 1000 m × 1000 m area and the sink node is deployed in the center. The random topology of these sensor nodes is shown in Figure 5. The communication range is 50 m and the initial energy is 2 J. The original data used in this section is synthetized of multiple data sets. Thus they cannot be sparsely represented in a predefined dictionary. To evaluate the energy consumption of ODL-CDG, we employ the same energy model in [31]:

(37) $$E_{trans}=\;\left\{ \begin{array}{l} l\;\times \;(E_{elec}+\;E_{mp}\;\times \;d^{2}),\;if\;d\;\leq \;d_{Thres}\\ l\;\times \;(E_{elec}+E_{mp}\;\times \;d^{4}),\;if\;d\;>\;d_{Thres} \end{array}\right.$$

(38) $$E_{rec}=l\times E_{elec}$$

where *E_trans_* denotes the energy consumption of transmitting *l* bits of data to another node within distance *d*, *E_rec_* denotes the energy consumption of receiving *l* bits of data, *E_elec_* denotes the energy consumption of the modular circuit, and *E_mp_* denotes the energy consumed by the power amplifying circuit. The parameters input to the ODL-CDG algorithm are the same as in Section 6.1 and the related parameters are listed in Table 2.

**Figure 6:**
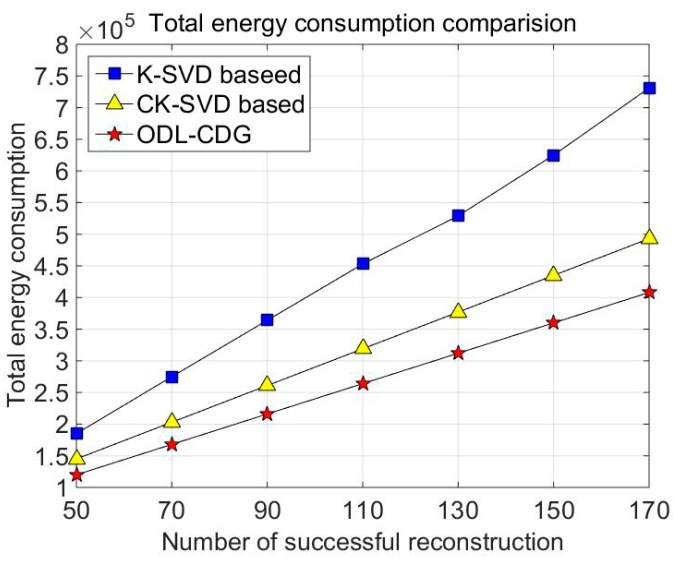
The total energy consumption of different dictionary learning based data gathering methods.

It is regarded as a successful reconstruction when the relative reconstruction error is smaller than 0.1. Figure 6 shows the energy consumption of ODL-CDG algorithm compared with other dictionary learning-based data gathering methods. Note that the K-SVD-based data gathering method requires one to access the whole data, so the original training data should be transmitted to the sink node by multi-hop paths. Moreover, the K-SVD dictionary should be updated in time since the synthetized data contains large diversities. Thus, the initial step of dictionary learning before data gathering may consume large amounts of energy. Therefore, the energy consumption of the K-SVD-based data gathering method is significantly larger than that of CK-SVD and the ODL-CDG. Figure 6 shows that ODL-CDG algorithm achieves the best energy savings. That’s because the dictionary in the ODL-CDG algorithm is learned in the process of compressive data gathering, which greatly reduces the energy consumption for raw data transmission through the entire network. Similarly, the total energy consumption of CK-SVD is enlarged with the increase of successful reconstruction number. But its energy consumption is still high than ODL-CDG as can be seen from Figure 6. The reason is that the introduced self-coherence penalty term can restrain the reconstruction error in the ODL-CDG algorithm, so the ODL-CDG algorithm should collect much fewer CS measurements than the CK-SVD-based data gathering method for the same reconstruction accuracy. 

**Figure 7:**
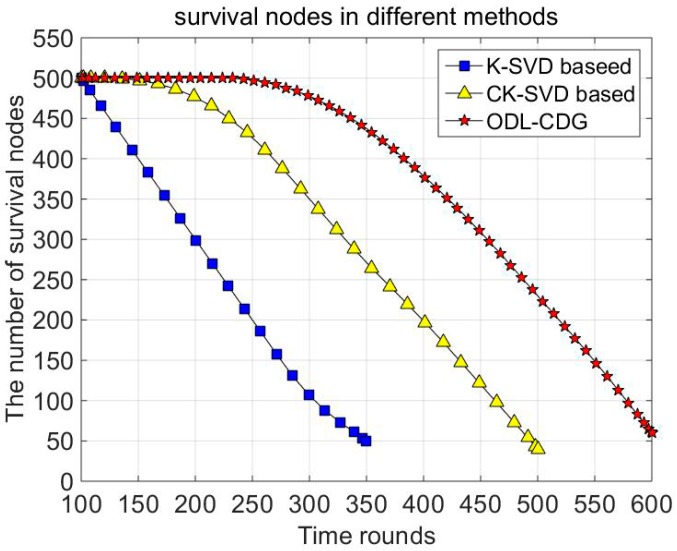
The number of survival nodes in different methods.

In Figure 7, the impact of different dictionary learning-based data gathering methods on the lifespan of nodes is studied. The node is supposed to survive when its energy is higher than zero. As can be seen from Figure 7, the ODL-CDG algorithm outperforms the other methods since the proposed dictionary has better adaptability to various signals. Thus the ODL-CDG algorithm reduces the energy consumption and prolongs the network lifespan.

## 7. Conclusions and Future Work

In this paper, we propose the ODL-CDG algorithm for energy efficient data collection in WSNs. The training signals for dictionary learning are obtained by a compressive data gathering approach, which greatly reduces the energy consumption. Inspired by the periodicity of natural signals, the learned dictionary is constrained with a sparse structure. To reduce the recovery error caused by environmental noise, the self-coherence of the learned dictionary is also introduced as a penalty term during the dictionary optimization procedure. Simulation results show the online dictionary learning algorithm outperforms both pre-specified dictionaries, like the DCT dictionary, and other dictionary learning approaches, like K-SVD, IDL and CK-SVD. The energy consumption of the ODL-CDG algorithm is significantly less than that of K-SVD-based and CK-SVD-based data gathering methods, which helps to enhance the network lifetime. In the future, we intend to employ other measurement matrices, such as the sparse measurement matrices, to further reduce the energy consumption. What’s more, how to apply the proposed algorithm to real large-scale WSNs is also a potential research direction.

## Figures and Tables

**Table 1:** Summary of notations.

*M*	Number of necessary measurements
*N*	Number of sensor nodes
*K*	Number of atoms of dictionary ***D***
*L*	Length of training data vectors
*λ*_A_	Sparse coefficient regularization parameter
*λ_Θ_*	Structured dictionary regularization parameter
*L_f_*	Lipschitz constant
***Φ***	Measurement matrix
***Ψ***	Orthonormal basis dictionary
***P***	Sensing matrix
***D***	Structured dictionary
***X***	Data matrix
$\hat{X}$	Estimated data matrix
***Θ***	Sparse atom representation dictionary

**Table 2:** Experimental parameters.

Parameter Name	Value
Node number	500
Transmission range	50 m
Initial energy	2 J
Data Size	1024 bit
*E_elec_*	50 nJ/bit
*E_amp_*	0.1 nJ/(bit·m^2^)
